# Dexamethasone Attenuates X-Ray-Induced Activation of the Autotaxin-Lysophosphatidate-Inflammatory Cycle in Breast Tissue and Subsequent Breast Fibrosis

**DOI:** 10.3390/cancers12040999

**Published:** 2020-04-18

**Authors:** Guanmin Meng, Melinda Wuest, Xiaoyun Tang, Jennifer Dufour, Todd P.W. McMullen, Frank Wuest, David Murray, David N. Brindley

**Affiliations:** 1 Department of Biochemistry, University of Alberta, Edmonton, AB T6G 2S2, Canada; guanmin@ualberta.ca (G.M.); xtang2@ualberta.ca (X.T.); 2 Cancer Research Institute of Northern Alberta, University of Alberta, Edmonton, AB T6G 2S2, Canada; mwuest@ualberta.ca (M.W.); Todd.Mcmullen@albertahealthservices.ca (T.P.W.M.); wuest@ualberta.ca (F.W.); David.Murray5@albertahealthservices.ca (D.M.); 3 Department of Oncology, Division of Oncologic Imaging, University of Alberta, Edmonton, AB T6G 2R7, Canada; jdufour@ualberta.ca; 4 Department of Surgery, University of Alberta, Edmonton, AB T6G 2R7, Canada; 5 Department of Oncology, Division of Experimental Oncology, University of Alberta, Edmonton, AB T6G 2R7, Canada

**Keywords:** adipocytes, adipose tissue, chemokines, cytokines, breast cancer, vasculitis

## Abstract

We recently showed that radiation-induced DNA damage in breast adipose tissue increases autotaxin secretion, production of lysophosphatidate (LPA) and expression of LPA_1/2_ receptors. We also established that dexamethasone decreases autotaxin production and LPA signaling in non-irradiated adipose tissue. In the present study, we showed that dexamethasone attenuated the radiation-induced increases in autotaxin activity and the concentrations of inflammatory mediators in cultured human adipose tissue. We also exposed a breast fat pad in mice to three daily 7.5 Gy fractions of X-rays. Dexamethasone attenuated radiation-induced increases in autotaxin activity in plasma and mammary adipose tissue and LPA_1_ receptor levels in adipose tissue after 48 h. DEX treatment during five daily fractions of 7.5 Gy attenuated fibrosis by ~70% in the mammary fat pad and underlying lungs at 7 weeks after radiotherapy. This was accompanied by decreases in CXCL2, active TGF-β1, CTGF and Nrf2 at 7 weeks in adipose tissue of dexamethasone-treated mice. Autotaxin was located at the sites of fibrosis in breast tissue and in the underlying lungs. Consequently, our work supports the premise that increased autotaxin production and lysophosphatidate signaling contribute to radiotherapy-induced breast fibrosis and that dexamethasone attenuated the development of fibrosis in part by blocking this process.

## 1. Introduction

About 60% of breast cancer patients receive breast-conserving surgery (lumpectomy) followed by radiation therapy (RT). Conventional RT for breast cancer involves irradiating the whole post-surgical breast with daily fractions of 1.8–2 Gy/fraction to a total dose of 45–50 Gy, although the most recent ASTRO guidelines for women with invasive breast cancer recommend hypo-fractionated RT with either 40 Gy in 15 fractions or 42.5 Gy in 16 fractions, i.e., ~2.6 Gy per fraction [1]. Fibrosis is a common late complication associated with RT [2,3,4]. For breast cancer patients, the frequency of RT-induced breast fibrosis is typically in the range of 15–28% [4,5,6]. Fibrosis progresses over subsequent decades and requires remediation with cosmetic surgery in severe cases [7]. RT-induced fibrosis also contributes to the development of lymphedema, which results in impaired function and decreased quality of life [8].

Increased signaling through the autotaxin (ATX)-lysophosphatidate (LPA)-LPA_1_ receptor pathway has been widely recognized as a major contributor to various types of fibrotic pathologies, notably of the liver and lungs [9,10,11,12,13,14,15,16,17,18,19,20]. In this pathway, the enzyme ATX generates LPA from lysophosphatidylcholine; LPA in turn activates six G-protein coupled LPA receptors that promote cell survival, proliferation and migration as well as inflammation [21]. Inhibition of ATX and LPA signaling significantly attenuated lung fibrosis in various disease models, including idiopathic pulmonary fibrosis (IPF) and the fibrosis that occurs following exposure to bleomycin (BLM) [19,22,23], a radiomimetic anticancer agent [24]. The key role for activation of this pathway in fibrosis was borne out in clinical trials where drugs such as GLPG1690 (an ATX inhibitor) and BMS986020 (an LPA_1_ receptor antagonist) attenuated the progression of IPF [25,26]. LPA-mediated pro-inflammatory signaling is also likely to contribute to RT-induced fibrosis, although this remains to be established.

Recent observations from our group have shown that ATX is an important factor in the biology of breast cancer as well as in its response to therapeutic intervention. We have further demonstrated a key role for the breast adipose tissue in these events. Notably, breast cancer cells produce very little ATX [27,28,29]. Instead, ATX is secreted by breast adipocytes and this activity increases in response to inflammation caused by cytokines produced by an adjacent breast tumor [30,31,32,33]. The resulting LPA is then able to activate LPA receptor-mediated signaling in the tumor cells. This bi-directional signaling between adipose tissue and breast tumors has been confirmed by other investigators [34,35]. Indeed, adipocytes produce ~40% of the body’s ATX [36,37] and the breast is rich in adipose tissue.

We subsequently showed that exposing cultured human breast adipose tissue to γ-radiation increased the secretion of ATX as well the expression of LPA_1_ and LPA_2_ receptors downstream of DNA damage [21]. These events activate a feed-forward inflammatory cycle since LPA increases the expression of cyclooxygenase-2 and multiple inflammatory cytokines/chemokines, which in turn promote further ATX secretion [32,38]. Similarly, in-vivo exposure of a breast fat pad of Balb/c mice to one 7.5-Gy fraction of RT increased plasma ATX concentrations as an early event; however, three daily fractions of 7.5-Gy were required to produce significant increases in the concentrations of various inflammatory cytokines/chemokines in plasma and breast tissue [39]. The presence of a syngeneic 4T1 breast tumor in the mammary fat pad also increased the levels of ATX and various inflammatory mediators in the adjacent mammary adipose tissue [40]. Exposure of the tumor and associated fat pad to fractionated RT further enhanced many of these responses, which were accompanied by increased infiltration of CD-45^+^ leukocytes into the tumor-associated adipose tissue [39]. 

We also reported [41] that an anti-inflammatory glucocorticoid, dexamethasone (DEX), produced a comprehensive suppression of LPA signaling in cultured human adipose tissue by decreasing the expression of ATX and of the LPA_1_ and LPA_2_ receptors. DEX also increased the expression of lipid phosphate phosphatase-1 (LPP1), an enzyme that degrades extracellular LPA and attenuates its ability to signal through LPA receptors [41]. As expected, these effects were accompanied by a decrease in the production of multiple inflammatory cytokines/chemokines [41]. The anti-inflammatory effects of glucocorticoids depend on: (a) binding of glucocorticoid receptors to glucocorticoid-responsive elements, which modify gene expression by inducing the expression of annexin I and MAPK phosphatase-1); (b) indirect effects on gene expression through the interactions of glucocorticoid receptors with transcription factors, including NFκB and activator protein 1; and (c) glucocorticoid receptor–mediated effects on second-messenger cascades including the PI3K–Akt–eNOS pathway [42]. These effects decrease the production of pro-inflammatory eicosanoids and cytokines, which would contribute to the observed attenuation of the activity of the ATX-LPA inflammatory cycle [33]. 

Based on the established ability of DEX to attenuate RT-induced inflammation of lung tissue [43,44,45] and lung fibrosis [45,46] and on our recent observation [41] that DEX suppresses ATX-LPA-LPA_1_ receptor signaling in cultured breast adipose tissue, we hypothesized that inhibiting the activation of the ATX-LPA-inflammatory cycle would attenuate the subsequent development of fibrosis in the irradiated breast in vivo. The potential involvement of ATX, LPA and the LPA_1_ receptor in such effects has not been evaluated previously, to our knowledge. Nor have the effects of DEX on RT-induced fibrosis of the breast. This is an important adverse side effect of RT that we anticipate to be particularly amenable to intervention in breast cancer because of the unique biology of the tumor-inflamed breast adipose tissue that was noted above. 

In the present study, we used normal Balb/c mice and the orthotopic 4T1-Balb/c mouse syngeneic model of breast cancer to examine the effect of DEX on LPA signaling and on fibrosis in the irradiated breast as well as in the underlying lung, which clinically can receive a significant dose of RT. The above-mentioned bidirectional cycle of ATX-LPA-inflammatory signaling between the adipose tissue and tumor is known to be established in this model [40]. We showed that DEX attenuated the RT-induced activation of the ATX-LPA-inflammatory cycle in cultured human adipose tissue after a single γ-ray exposure and also in vivo in a breast fat pad of female mice after fractionated X-ray exposures. We also showed that DEX treatment during fractionated RT attenuated fibrosis in the irradiated fat pad of Balb/c mice by ~70% at 7 weeks following the irradiation. This study is the first to demonstrate that inhibiting aspects of the RT-induced activation of the ATX-LPA-inflammatory cycle is a potential strategy for decreasing RT-induced breast fibrosis. In addition, we showed that DEX attenuated inflammation and fibrosis in the lungs caused by coincidental irradiation, as expected based on work by other investigators [43,44,46]. However, our work uniquely links this activity of DEX to its ability to attenuate LPA signaling, which is now recognized as a major stimulus for the progression of fibrosis.

## 2. Results

### 2.1. DEX Attenuates the Activation of the ATX-LPA-Inflammatory Axis in Breast Adipose Tissue and Tumor during RT

**Figure 1:**
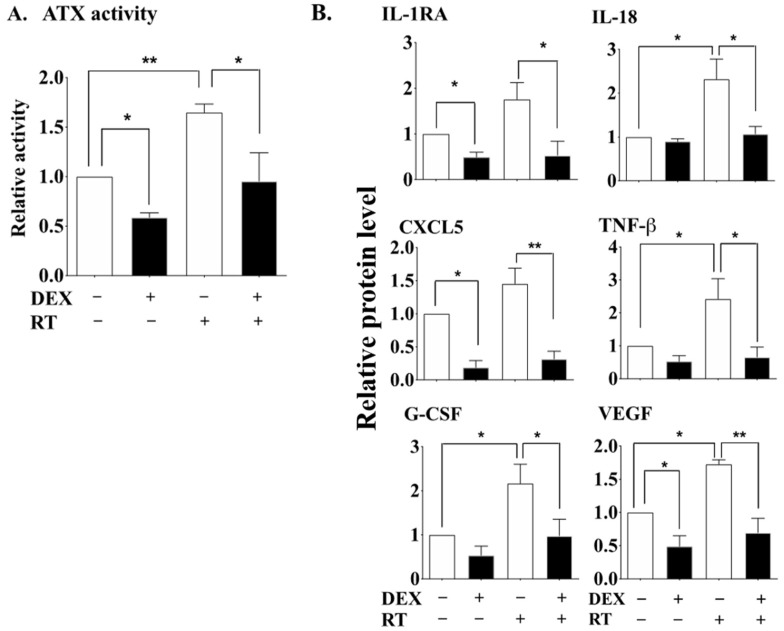
DEX attenuated the radiation-induced increase in ATX, cytokines and chemokines in human adipose tissue. Cultured human adipose tissue was treated with 100 nM DEX for 24 h prior to exposure to 1 Gy of γ-radiation. After 48 h, ATX activity (**A**) and the levels of cytokines/chemokines (**B**) were measured in the culture medium. Results from 6 paired adipose tissue samples were expressed as means ± SEM relative to the non-irradiated tissue. * *p* < 0.05 and ** *p* < 0.01. Absolute values are similar to those published previously [41].

We demonstrated previously that the anti-inflammatory glucocorticoid, DEX, has a coordinated action in regulating the ATX-LPA-inflammatory cycle [41]. We therefore investigated whether DEX could inhibit the RT-induced activation of the ATX-LPA-inflammatory cycle by using a dose of 1 Gy of γ-rays with cultured human adipose tissue as established previously [21]. An optimum concentration of 100 nM DEX [41] attenuated the effects of irradiation in increasing ATX activity in the culture medium (Figure 1A) and also decreased ATX in the absence of irradiation, as expected [41]. 

DEX also abrogated the RT-induced increases in the concentrations of several cytokines and chemokines, including IL-18, TNFβ, G-CSF and VEGF (Figure 1B). These mediators are involved in immune responses including activation of various leukocytes and also in the stimulation of angiogenesis. The apparent increases for IL-1RA and CXCL5 after irradiation were not statistically significant, but DEX decreased the concentrations of both of these proteins in irradiated and non-irradiated samples. The DEX-induced decrease in IL-1RA was unexpected since IL-1RA should be anti-inflammatory and corticoid steroids are expected to increase its expression [47].

We validated these results by determining the effects of DEX on RT-mediated events in vivo using normal mice and also mice with orthotopic 4T1 breast tumors. Both mouse models were treated daily with 3 mg/kg DEX or vehicle [41] on the day before RT, during the irradiation of a mammary fat pad with 7.5 Gy of X-rays for three consecutive days, and on the day after the completion of RT. DEX treatment did not significantly alter the RT-induced decrease in tumor weight or tumor volume (Meng, G. and Brindley, D. N., University of Alberta, Edmonton, AB, Canada). 

**Figure 2:**
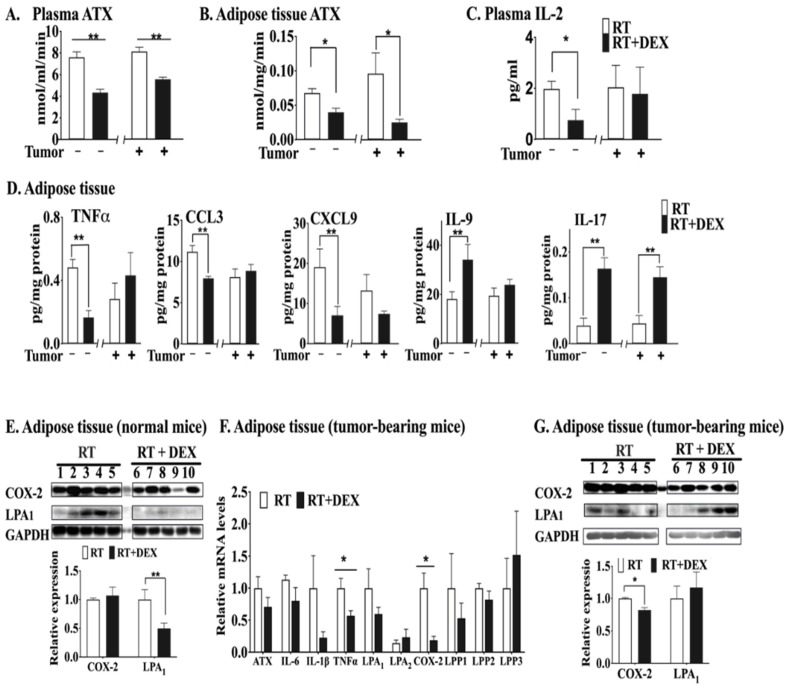
DEX attenuated the RT-induced activation of the ATX-LPA-inflammatory cycle in the breast adipose tissue of both normal (i.e., non-tumor-bearing) mice and tumor-bearing mice. Both tumor-bearing and normal mice were treated daily with 3 mg/kg DEX or vehicle 1 day before RT, during the exposure of a mammary fat pad to 3 daily 7.5-Gy fractions of X-rays, and 1 day after RT. At 48 h after completion of the RT, samples from both mouse models were analyzed for: (**A**) ATX activity in plasma; (**B**) ATX activity in mammary adipose tissue; (**C**) IL-2 in plasma; (**D**) cytokines/chemokines in mammary adipose tissue; (**E**) COX-2 and LPA_1_ receptor protein levels determined by Western blot analysis in mammary adipose tissue of normal mice after treatment with RT alone (mice 1–5) or after RT with DEX treatment (mice 6–10); (**F**) mRNA levels of ATX, IL-6, IL-1β, TNFα, LPA_1/2_ receptors, COX-2, LPP1, LPP2 and LPP3 in tumor-adjacent adipose tissue; (**G**) COX-2 and the LPA_1_ receptor protein levels in the tumor-adjacent adipose tissue determined by Western blot analysis after treatment with RT alone (mice 1–5) or after RT with DEX treatment (mice 6–10);. White columns indicate no DEX treatment and black columns indicate DEX treatment. Results are expressed as means ± SEM with *n* = 5 mice/group. * *p* < 0.05 and ** *p* < 0.01. More detailed Western blots are shown in Figure S1A.

DEX significantly decreased plasma ATX activity after RT in both mouse models (Figure 2A). The basal ATX activity in the mammary adipose tissue of tumor-bearing mice was higher (Figure 2B) than that in the normal mice, since breast tumors cause inflammation, which increases ATX production [28]. DEX also effectively decreased ATX activity at 48 h after three 7.5-Gy fractions of RT in the mammary adipose tissue of normal mice and of tumor-bearing mice (Figure 2B). In terms of inflammation, DEX decreased the concentration of IL-2 in plasma of normal mice treated with RT, but not in tumor-bearing mice (Figure 2C). Combining DEX with RT also decreased the concentrations of pro-inflammatory TNFα, CCL3 and CXCL9 in irradiated adipose tissue of normal mice. Conversely, treating normal mice with DEX increased the levels of IL-9 and IL-17 (Figure 2D), cytokines which are reported to mediate anti-inflammatory effects [48,49]. The only cytokine that was significantly increased by irradiation of adipose tissue in tumor-bearing mice was IL-17. Western blot analysis of the irradiated adipose tissue from normal mice showed that DEX treatment had no significant effect on COX-2 expression, but it did decrease the concentration of LPA1 receptor protein (Figure 2E). mRNA levels of TNFα and COX-2 in irradiated adipose tissue adjacent to the tumor were decreased by DEX in tumor-bearing mice (Figure 2F). The apparent decrease for LPA1 receptor mRNA did not reach the level of significance and there was no significant effect on the level of LPA1 receptor protein (Figure 2G). The protein level of COX-2 in irradiated breast adipose tissue of tumor-bearing mice was decreased by DEX.

**Figure 3:**
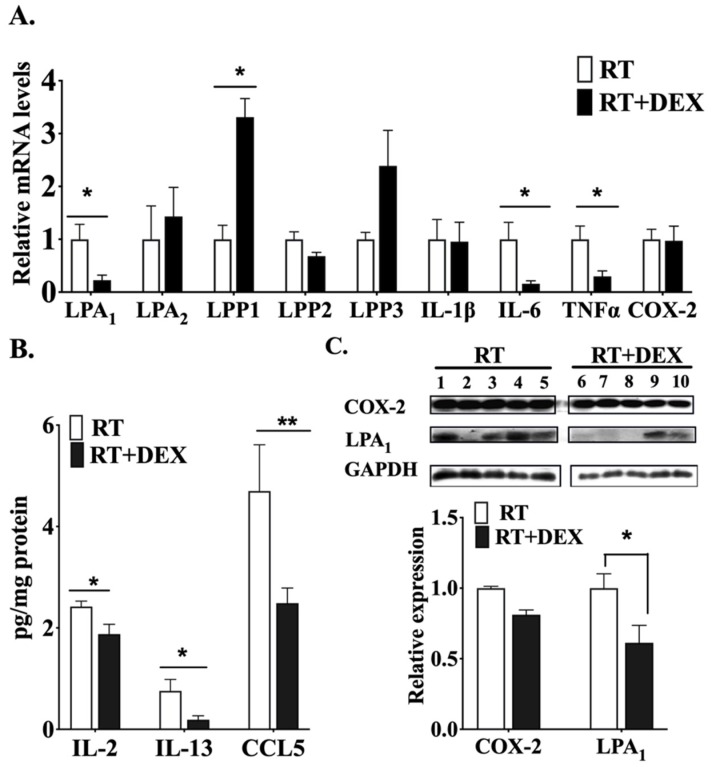
DEX attenuated the RT-induced activation of the ATX-LPA-inflammatory cycle in the tumor tissue. Mice bearing 4T1 breast tumors were treated daily with DEX (3 mg/kg) or vehicle 1 day before RT, during the exposure of a mammary fat pad to 3 daily 7.5-Gy fractions of X-rays, and 1 day after RT. At 48 h after completion of the RT the tumor tissue was analyzed for: (**A**) mRNA levels of LPA_1/2_ receptors, LPP1, LPP2, LPP3, IL-1β, IL-6, TNFα and COX-2; (**B**) concentrations of cytokines/chemokines determined by Multiplex ELISA; and (**C**) COX-2 and the LPA_1_ receptor protein levels determined by Western blot analysis. White columns indicate no DEX treatment and black columns indicate DEX treatment. Results are expressed as means ± SEM with 5 mice/group. * *p* < 0.05, ** *p* < 0.01. More detailed Western blots are shown in Figure S1B.

mRNA levels of inflammatory cytokines in tumor tissue of irradiated mice were also modulated by DEX, showing a pronounced decrease for IL-6 and TNFα (Figure 3A). DEX also decreased the mRNA for LPA1, while there was a ~3 fold increase for LPP1, which degrades LPA. Concentrations of IL-2, IL-13 and CCL5 in the irradiated tumor were similarly decreased by DEX treatment (Figure 3B). LPA1 receptor protein expression in irradiated tumors was also decreased by DEX, although there was no significant change in COX-2 in the tumors (Figure 3C).

These combined results demonstrate that DEX decreases the potential for LPA signaling and the inflammatory environment produced by RT in breast adipose tissue and breast tumors.

### 2.2. Dexamethasone Attenuates RT-Induced Fibrosis at 7 Weeks in the Mammary Fat Pad and in the Underlying Lung

So far, our results show that DEX attenuates the RT-induced activation of the ATX-LPA-inflammatory cycle in cultured human adipose tissue and in mice where the mammary fat pad was irradiated. We next studied if DEX treatment affects RT-induced fibrosis by irradiating the left 2nd mammary fat pad of normal female mice with 5 daily fractions of 7.5 Gy. Half of the mice were treated with 3 mg DEX/kg at 1 day before the RT, daily during the 5 days of RT and for 3 days after completion of RT to control ATX production and inflammation after the RT. Control mice were treated at the same times with vehicle. Fibrosis was measured at 7 weeks after completion of the RT by staining sections of the irradiated fat pad and the underlying lungs with Sirius Red for collagen and Masson’s trichrome for connective tissue. We also determined the collagen content of the inguinal fat pad, which was not irradiated. As additional controls, sections of the irradiated fat pad and lungs were taken at 2 days after completion of the 5-day RT regimen, i.e., before fibrosis was expected to develop. Non-tumor-bearing mice had to be used for these studies because the 4T1 model produces extensive lung metastasis in <3 weeks from the injection of cancer cells and the mice would not survive long enough to develop fibrosis.

**Figure 4:**
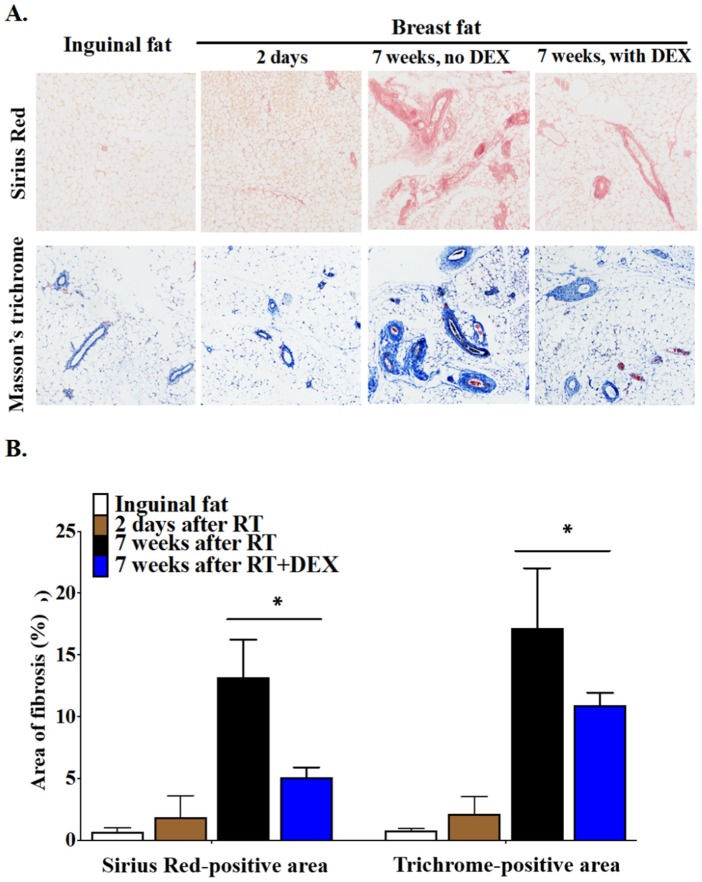
DEX decreased RT-induced fibrosis in the breast fat pad of mice at 7 weeks post-irradiation. Balb/c mice were treated daily with 3 mg/kg DEX at 1 day before RT, during 5 daily 7.5-Gy fractions of RT to an upper breast fat pad, and for 3 days after RT. Control mice were injected with vehicle. (**A**) At 7 weeks after completion of the RT, fibrosis in sections of the fat pad was measured by collagen staining with Sirius Red (collagen stained in red) and Masson’s trichrome staining (collagen stained in blue). The corresponding inguinal fat pad, which was not irradiated, was used as a control. As an additional control, we stained the fat pad on day 2 after completion of the RT, which was before fibrosis could develop. Magnification: 100×; (**B**) Quantification of fibrosis by Sirius Red or Masson’s trichrome staining was conducted on 5-8 different fields and results were averaged for each mouse (*n* = 6 mice/group). * *p* < 0.05.

The irradiated breast fat pad showed extensive fibrosis at 7 weeks post-RT (Figure 4). Adipocytes appeared to be intact (Figure 4A). Thick collagen fibers were widely deposited around blood vessels, forming a tangled composition within the cellular intervals that disrupted the continuity of the adipose tissue (Figure 4A). DEX treatment decreased RT-induced fibrosis by ~70% at 7 weeks post-RT, with the fibers being thinner as compared to tissue from mice not treated with DEX (Figure 4A,B). Staining with Masson’s trichrome also revealed marked interstitial and perivascular fibrosis in the irradiated breast fat tissue at 7 weeks after RT, and this was attenuated by ~50% by DEX treatment. 

**Figure 5:**
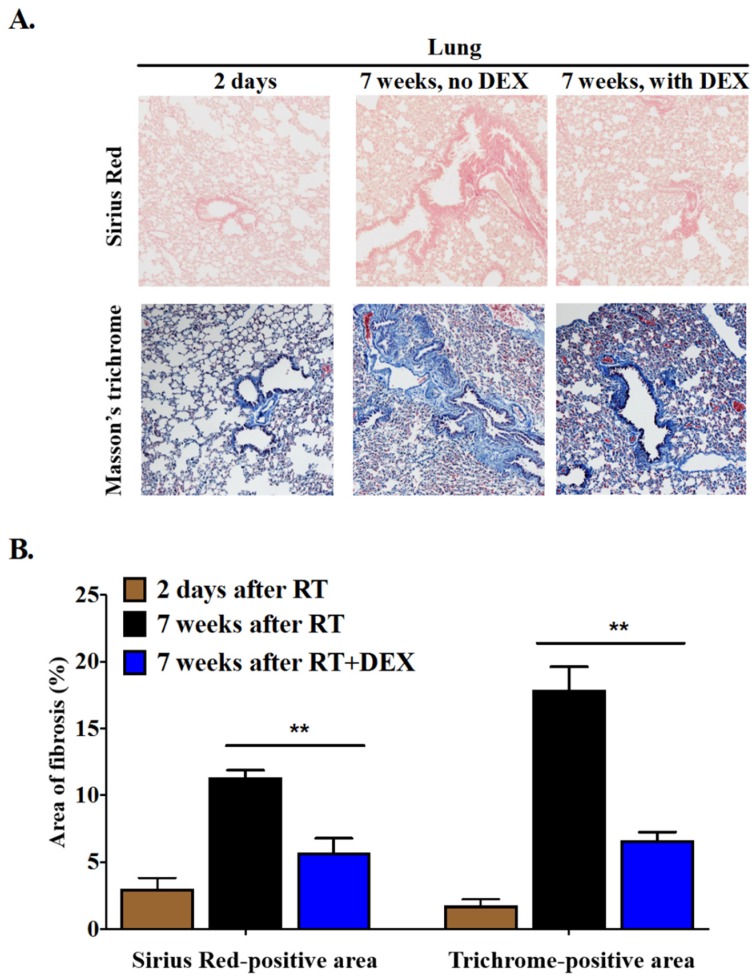
DEX decreased RT-induced fibrosis in the lung. Mice were treated daily with 3 mg/kg DEX at 1 day before RT, during 5 daily 7.5-Gy fractions of RT to an upper breast fat pad, and for 3 days after RT. Control mice were injected with vehicle. (**A**) Lung sections underlying the irradiated area were stained for collagen with Sirius Red and Masson’s trichrome at 7 weeks after completion of the RT. The control lung tissue was obtained on day 2 after the last RT before fibrosis could develop. Magnification: 100×; (**B**) Quantification of fibrosis determined by Sirius Red or Masson’s trichrome staining was conducted on 5–8 different fields and results were averaged for each mouse (*n* = 6 mice/group). ** *p* < 0.01.

Although RT was focused on the breast fat pad, we also expected to see RT-induced fibrosis in the underlying lungs because of coincidental RT exposure. Indeed, a significant degree of fibrosis was observed in the irradiated lung tissue (Figure 5). DEX treatment decreased lung fibrosis by ~70% as measured by staining with Sirius Red or Masson’s trichrome compared to the 2-day post-RT control.

**Figure 6:**
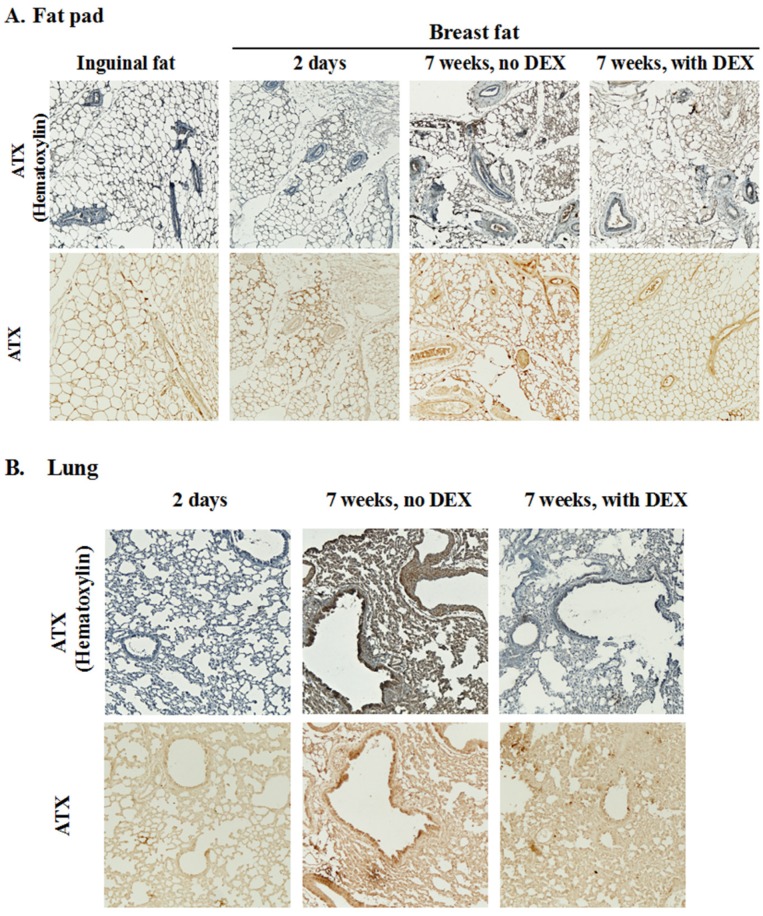
DEX attenuated the RT-induced association of ATX with blood vessels in mammary adipose tissue and lung. Mice were treated as in Figure 5. Representative images from six mice/group of the immunohistochemistry are shown for ATX expression (brown) in the mammary fat pad (**A**) and lung sections (**B**) at 7 weeks after completion of the RT. Magnification: 100×. The upper images in each panel are for tissue sections counterstained with hematoxylin (blue-purple) to show morphology.

DEX treatment decreased plasma ATX activity at two days after three fractions of RT by ~43% in normal mice (Figure 2A); however, plasma ATX activity returned to the non-DEX treatment level at 7 weeks after five fractions of RT (Figure S2A). Total ATX activities in the irradiated fat pad and lungs were also not changed significantly after 7 weeks by prior treatment with DEX (Figure S2B). We also determined the distribution of ATX in the adipose tissue and lungs (Figure 6). ATX was evenly distributed in the interstitium of adipose tissue in both the control mammary fat pad (i.e., 2 days post-RT) and the inguinal fat pad and it was barely expressed around or within blood vessels. The distribution pattern of ATX was altered at 7 weeks after RT when ATX was localized around the vessels. This distribution aligned with the fibrotic areas in fat pads and lungs (Figure 4 and Figure 5). The association of ATX with blood vessels was attenuated by DEX (Figure 6) along with the decreased fibrotic response.

**Figure 7:**
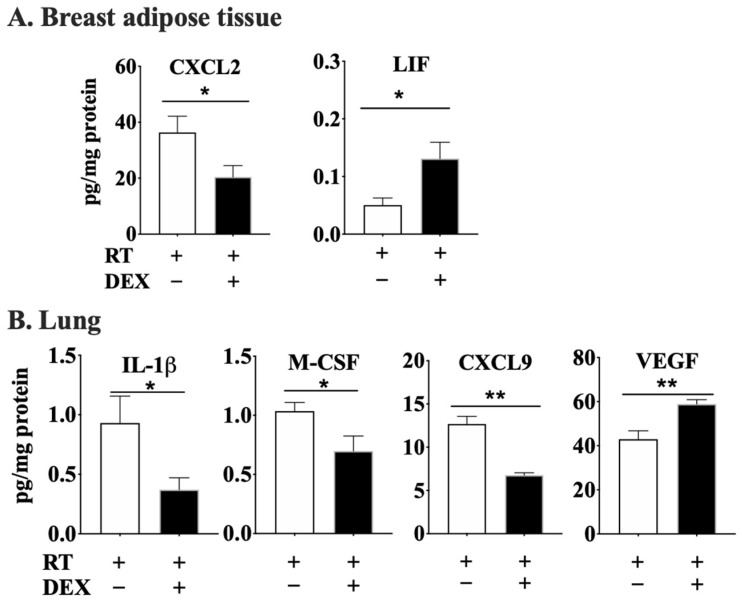
DEX attenuated the RT-induced effects on the concentrations of cytokines, chemokines and growth factors in mammary adipose tissue and lung tissue at 7 weeks post-RT. Mice were treated as in Figure 5. At 7 weeks after completion of the RT, the irradiated adipose tissue (**A**) and lung (**B**) were collected for multiplex cytokine analyses. Results are expressed as means ± SEM from six independent experiments. * *p* < 0.05, ** *p* < 0.01.

DEX treatment at the time of RT decreased the concentration of CXCL2 and increased that of LIF in the adipose tissue after 7 weeks (Figure 7A). DEX treatment at the time of RT also decreased the concentrations of IL-1β, M-CSF and CXCL9 in lung at 7 weeks post-RT, although VEGF concentrations were increased (Figure 7B).

**Figure 8:**
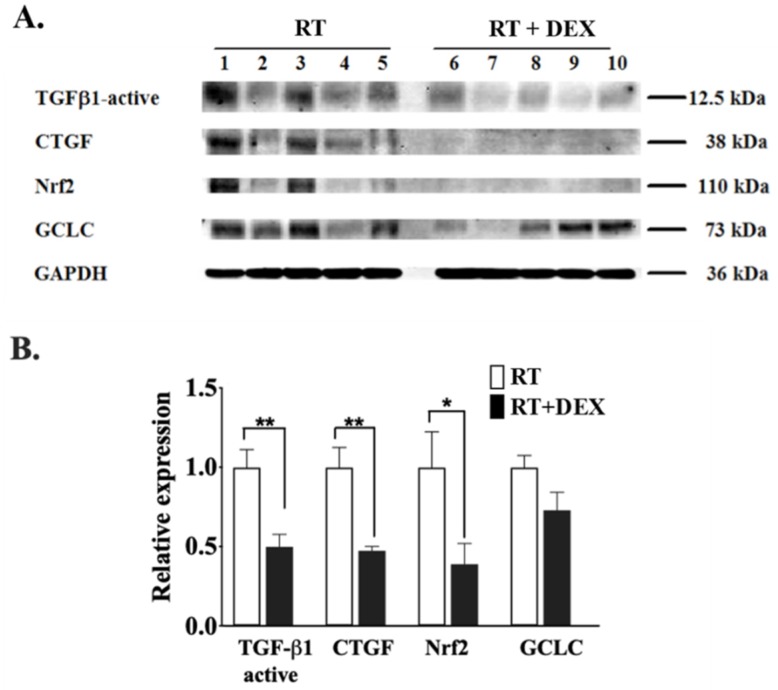
DEX decreased the levels of activated TGF-β1, CTGF and Nrf2 in irradiated mammary adipose tissue. Mice were treated as in Figure 5. (**A**) Protein levels of active TGF-β1, CTGF, Nrf2 and GCLC were determined by Western blot analysis at 7 weeks after completion of the RT for five mice that were not treated (1–5) or were treated with DEX (6–10), respectively. The estimated molecular weight of the protein is shown. (**B**) Results are expressed as means ± SEM with five mice/group. White columns indicate no DEX treatment and black columns indicate DEX treatment. * *p* < 0.05, ** *p* < 0.01. More detailed Western blots are shown in Figure S1C.

We also studied the effect of DEX treatment on the pro-fibrotic cytokine, transforming growth factor β (TGF-β), which has a well-known role in the pathogenesis of RT-induced fibrosis of the lung and other tissues in both rodent models and humans [50,51,52,53,54]. We focused on TGF-β1 since it is the most ubiquitously distributed isoform in mammalian tissues and it contributes largely to the pro-fibrotic effects of TGF-β. Although there was considerable variability among the individual animals, the levels of the active form TGF-β1 was clearly decreased by DEX at 7 weeks after five fractions of RT in adipose tissue (Figure 8A,B). Connective tissue growth factor (CTGF), a downstream effector of TGF-β and another key mediator of the fibrotic cascade [53,54], was also decreased by DEX.

We indirectly assessed the effect of DEX on oxidative stress, another recognized mediator of fibrosis [2,53], in our model system by monitoring the level of the antioxidant transcription factor nuclear factor erythroid 2-like factor 2 (Nrf2), which is modulated by ionizing radiation [55]. Nrf2 levels in the irradiated breast adipose tissue at 7 weeks post-RT were decreased by DEX, whereas the apparent decrease of its downstream target glutamate-cysteine ligase catalytic subunit (GCLC) did not reach the level of statistical significance (Figure 8A,B).

## 3. Discussion

The primary focus of this study was on the potential role of LPA signaling in promoting fibrosis in the context of the treatment of breast cancers with RT, where fibrosis of the breast tissue represents a major adverse side effect. About 15–28% of breast cancer patients develop Grades 1 and 2 fibrosis in the irradiated breast at three years after RT [4,5,6]. This fibrosis progresses for decades [7] and it requires cosmetic correction in severe cases. Our secondary focus was on the role of LPA signaling in RT-induced fibrosis and its amelioration in the underlying lung tissue. Even with state-of-the-art RT treatment planning and delivery, post-operative whole-breast RT for breast cancer results in a significant dose of radiation to a portion of the lung, which can lead to adverse events such as pulmonary fibrosis or to losses of 10–15% in pulmonary function [56]. These effects can be tolerated because of excess lung capacity, but they could be minimized if the underlying mechanisms were better understood and exploited.

In this study we therefore tested the hypothesis that an anti-inflammatory agent, DEX, would attenuate the development of RT-induced fibrosis in the breast and further that the inhibition of LPA signaling by DEX would contribute to such an effect. We also measured the attenuation of fibrosis of the lung, in part because of its above-mentioned clinical relevance, but also because there is a significant literature describing the ability of DEX to attenuate acute inflammatory responses (early pneumonitis) and longer-term fibrosis in the irradiated lung [43,44,45,46]. These earlier studies did not consider a potential role for ATX-LPA signaling in the observed effects. However, studies of idiopathic pulmonary fibrosis and of lung fibrosis induced by the anticancer drug, bleomycin, which is widely used as a model for IPF, have shown an important role for ATX and LPA signaling [19,57]. BLM is also a DNA-damaging radiomimetic agent [24] and thus it should partially model the effects of ionizing radiation the lung.

We first showed that DEX effectively inhibited RT-induced activation of the ATX-LPA-inflammatory cycle in irradiated cultures of human (breast- and neck-derived) adipose tissue following a single γ-ray exposure. Next, we demonstrated that similar effects of DEX occurred in vivo in normal female Balb/c mice following delivery of fractionated RT (3 × 7.5 Gy X-rays) to a single mammary breast fat pad. Treatment of mice with DEX daily from one day before until one day after RT suppressed various aspects of the RT-mediated activation of LPA signaling and inflammation in the adipose tissue of normal mice, including attenuating the increases in ATX, LPA_1_ receptors, TNFα, CCL3 and CXCL9 at two days after completion of the RT. Similar effects were seen in syngeneic 4T1 breast tumor-bearing mice where the presence of the tumor increased inflammation of the breast fat pad [40]. In this case, the additional inflammatory effects of RT on tissue cytokine/chemokine levels were muted.

The effects of 100 nM DEX in decreasing ATX activity in adipose tissue were related to a decrease in mRNA expression for ATX and not to a direct inhibition ATX activity by DEX [41]. This conclusion is supported by the observation that although bile salts with hydroxyl substitution at position 7 of the steroid moiety bind to ATX with an IC_50_ of ~10 µM and cause inhibition, DEX did not have an inhibitory effect [58]. 

We then studied RT-induced fibrosis in the irradiated breast fat pad and the underlying lung tissue of normal mice that were treated with DEX or vehicle daily from 1 day before until 3 days after RT. DEX attenuated fibrosis at 7 weeks post-RT in the irradiated mammary adipose and underlying lung tissue by ~70%. At this time, DEX treatment resulted in a decrease in the concentration of CXCL2 in the irradiated adipose tissue and of IL-1β, M-CSF and CXCL9 in the lung. These effects of DEX on inflammatory cytokines and chemokines, which promote fibrosis [59], are consistent with its observed anti-fibrotic activity. DEX also increased VEGF concentrations in the lungs and LIF in adipose tissue, and these proteins are known to attenuate fibrosis [60,61]. It is significant that although ATX activity in adipose tissue was not increased at 7 weeks post-RT (Figure S2), there was a clear change in ATX distribution (Figure 6). At this time, ATX accumulated in the vicinity of blood vessels, which coincided with the major site of fibrosis. This is significant in ATX signaling because it is thought that the bulk levels of ATX are much less important than the specific binding of ATX to the cell surface through integrins and syndecan-4, which channels LPA signaling to its receptors [62,63,64]. DEX attenuated this perivascular accumulation of ATX, which is probably part of the mechanism for the observed decrease in fibrosis. Further work is required to establish the direct link between the decreased perivascular accumulation of ATX and fibrosis. However, recent studies have shown that ATX-LPA signaling through LPA_1_ receptors plays a critical role in vascular development [65] and vasculitis [66,67]. Increased LPA signaling is also associated with injury to the vascular endothelium, including the enhanced vascular permeability/leakage seen in BLM-induced lung fibrosis in mice, where inhibiting signaling through LPA_1_ receptors attenuated both vascular injury and fibrosis [22,57,68,69]. Inhibition of ATX accumulation in the perivascular area by DEX could thus contribute to the observed attenuation of fibrosis by decreasing local LPA signaling. Indeed, injury to the vascular endothelium (such as enhanced permeability and decreased blood flow) has been suggested to contribute to RT-induced fibrosis in animal models and this is known to be modulated by DEX [70,71,72].

We used normal animals in the fibrosis experiments for two reasons. First, this is standard practice in studies of normal tissue complications post-RT, including earlier studies of the modulation of lung injury by DEX [43,44,45,46]. Second, as noted above, the highly aggressive nature of the 4T1 tumors precluded such long-term studies in tumor-bearing animals. We should note, however, that the unique biology of the breast, in which bi-directional crosstalk between the tumor and adipose tissue increases the level of inflammation, ATX secretion and signaling through the ATX-LPA pathway, means that RT-induced fibrosis in the diseased breast should be even more susceptible than the normal breast to attenuation by agents such as DEX. Although clinically the tumor is normally resected about 3–4 weeks prior to RT, the post-surgical breast at this time is probably still residually inflamed – not only from the influence of the initial tumor and residual tumor cells but also potentially from the surgical procedure itself.

Numerous studies employing genetic or pharmacological modulation of the ATX-LPA pathway in mice treated with the radiomimetic drug BLM have established an important role for LPA signaling through the LPA_1_ receptor in the DNA damage-mediated fibrosis of organs such as lung [14,19,22,23,57,73,74]. For example, the ATX inhibitor GWJ-A-23 attenuated BLM-induced pulmonary fibrosis as well as the levels of LPA and TGF-β in the lung [14]. A similar role for LPA signaling in the pathogenesis of IPF is apparent from the elevated levels of LPA seen in the lungs of affected individuals (e.g., [22]). Clearly, the ATX-LPA-LPA_1_ axis is strongly implicated in different types of fibrosis in various tissues, notably the lung. By extension, and given the comprehensive effects of DEX on this pathway reported in our earlier [41] and present studies, it follows that the ATX-LPA pathway could also contribute directly to the fibrosis of breast and lung tissue seen after RT exposure.

There are no published studies that to our knowledge have looked at the role of ATX-LPA signaling in the context of RT-induced fibrosis of the breast. However, Gan and colleagues [12] showed that a selective LPA_1/3_ antagonist, VPC12249, lowered the levels of the TGF-β1 and CTGF pro-fibrotic cytokines in irradiated mouse lung, as well as attenuating RT-induced fibroblast accumulation, myofibroblast activation, collagen deposition and fibrosis therein. These findings suggest that LPA-LPA_1/3_ signaling through CTGF may contribute significantly to RT-induced fibrosis of the lung. Such a scenario is consistent with the study by Tager and colleagues [22] showing that the LPA_1_ receptor plays a similarly important role in BLM-induced lung fibrosis.

As noted above, TGF-β is probably the most widely studied pro-fibrotic factor that has a clear relationship to RT-induced collagen deposition and lung fibrosis [2,3,53,54]. Indeed, TGF-β plays a critical role in every stage of RT-induced fibrosis, including its initiation, development, and persistence [75]. As expected, we observed that DEX decreased the level of active TGF-β in irradiated breast adipose tissue at 7 weeks post-RT, and this was accompanied by a decrease in the levels of CTGF, secretion of which is stimulated by TGF-β [76]. So how might ATX-LPA signaling fit into the overall pathway leading to RT-induced breast fibrosis? Again, the above-mentioned substantial body of data relating to BLM-induced fibrosis of the mouse lung appears relevant to this consideration. A recent model [52] posits that the increased levels and activity of ATX in the lung after BLM treatment leads to increased LPA levels/signaling and thus to downstream events such as fibroblast recruitment, survival, proliferation and conversion to myofibroblasts (which mediate the excessive deposition of extracellular matrix) [53], epithelial cell death leading to pro-inflammatory cytokine production, and vascular endothelial permeabilization leading to vascular leakage. LPA receptors also signal through TGF-β, likely involving the intermediacy of integrins, notably αvβ6, which can bind to and activate TGF-β from its latent form, a pathway that likely plays a key role in the inflammation and fibrosis of the lung [52,57,77,78].

Our earlier studies of cultured human breast adipose tissue exposed to γ-rays showed that inhibiting ATR, PARP-1 or NFκB attenuated the radiation-induced activation of the ATX-LPA-inflammatory cycle [21]. On this basis, it is tempting to speculate that the ATX-LPA axis connects the upstream DNA damage response to key downstream pro-fibrotic events such as activation of TGF-β and CTGF. In addition, the ATX-LPA axis cross-talks with signaling through sphingosine 1-phosphate, the sphingolipid analogue of LPA. Treatment of mice with DEX decreases plasma concentrations of both LPA and sphingosine 1-phosphate [41] and this combined action could contribute to decreased signaling and cross-talk with the production of pro-inflammatory cytokines such as TGF-β and CTGF that promote fibrosis [79].

In summary, several publications have shown that DEX is effective in attenuating RT-induced inflammation and fibrosis in the mouse lung [43,44,45,46]. We should note that in lungs, ATX is produced by bronchial epithelial cells [80,81]. Our results support the findings concerning lung fibrosis and extend them to fibrosis of the breast tissue where ATX is produced primarily adipocytes. The present studies provide the first “proof-of-principle” that inhibiting the ATX-LPA signaling axis and the subsequent post-RT cytokine surge may provide a valid approach to decreasing late morbidity from breast fibrosis. We are currently examining this hypothesis using other more specific inhibitors of the ATX-LPA-inflammatory cycle, which may have fewer adverse side effects including the suppression of immune responses. If these effects prove to be temporally robust, unlike those seen with DEX and other glucocorticoids in RT-induced lung inflammatory mediator responses, pneumonitis and fibrosis in rodent models [44,82,83], then this approach may be especially relevant in the treatment of breast cancer with RT since adipocytes are the major source of ATX in the breast. Multiple fractions of RT to the whole breast after lumpectomy should stimulate ATX production from adipocytes, promoting LPA production and signaling through the concomitantly increased levels of LPA_1_ receptors after RT [39]. These effects of LPA activate the inflammatory cycle and drive the progression of fibrosis.

## 4. Materials and Methods

### 4.1. Reagents

DEX (≥97%) was purchased from Sigma-Aldrich (St. Louis, MO, USA). Penicillin/streptomycin and Roswell Park Memorial Institute (RPMI) 1640 medium were from Gibco/Life Technologies (Burlington, ON, Canada). Amplex^TM^ Red was from Invitrogen Life Technologies (Camarillo, CA, USA). Antibodies against LPA_1_ and CD45 were from Abcam (Cambridge, UK), and cyclooxygenase-2 (COX-2) antibody was from Santa Cruz Biotechnology, Inc. (Santa Cruz, CA, USA). HRP conjugated anti-rabbit IgG antibody and DAB was from DAKO (Carpinteria, CA, USA). All PCR primers were purchased from Integrated DNA Technologies Inc. (Coralville, IA, USA). Other chemicals and reagents were from Sigma-Aldrich unless indicated otherwise.

### 4.2. Culture of Human Adipose Tissue and Radiation Exposure

Samples of human adipose tissue were obtained with approval of the University of Alberta Health Research Ethics Board ID Pro00018758 with written informed consent as described previously [41]. Adipose tissue was minced into small pieces (~1–2 mm^3^) under sterile conditions. Minced samples were incubated in 1 mL RPMI 1640 serum-free medium containing 0.1% (w/v) fatty acid–free bovine serum albumin. After an overnight incubation, adipose tissues were incubated with 100 nM DEX or control medium for 24 h before exposure to 1 Gy of γ-radiation [21]. The medium was then replaced with fresh medium and DEX and the tissue was incubated for a further 48 h. 

### 4.3. Measurements of the Effects of DEX on the RT-Induced Inflammatory Responses of Tumor-Bearing and Normal (i.e., Non-Tumor-Bearing) Mice 

Female Balb/c mice aged 8–10 weeks old were from Charles River (Kingston, ON, Canada) and were housed according to guidelines of the Canadian Council on Animal Care as approved by the University of Alberta/Cross Cancer Institute Animal Welfare Committees (AUP00000226 and AC16223). Mice were maintained at 21 ± 2 °C, 55 ± 5% humidity and a standard 12-h light-dark cycle. Mice had free access to standard laboratory diet (4% fat) and water. A syngeneic orthotopic model of breast cancer was chosen using Balb/c mice that were injected into the left 2nd mammary fat pad with 20,000 4T1 breast cancer cells [29]. 4T1 tumors were allowed to grow for 10 days when tumor sizes reached ~3 × 3 to 4 × 4 mm before RT. We irradiated the mammary fat pad of normal mice with X-rays using an image-guided small-animal radiation research platform (SARRP; Xstrahl Inc., Camberley, UK). This was achieved using a 10 × 10 mm square collimator with two beams at -45° and +45° angles. Each beam duration was ~80 sec. The isocenter was defined using a CT dot placed on top of the mammary fat pad. When a tumor was present in the mammary fat pad, we also used a 10 × 10 mm square collimator with two beams between −25° to −45° and +25° to +45° angles. These parameters depended on the size of the tumor over the course of the irradiation period. The isocenter was defined in the middle of the 3-D tumor volume.

For determination of the effects of DEX on the responses to RT, Balb/c mice or mice with a 4T1 tumor were treated daily with 3 mg/kg DEX [41] i.p. or vehicle (0.1% ethanol in PBS) for 5 days. The DEX treatments were administered at 1 day before RT, at 1–2 h prior to each of the three daily 7.5 Gy fractions of RT to the breast fat pad or to the fat pad with the tumor, and at one day after the final fraction of RT. The mice were anesthetized terminally at two days after completion of the RT by i.p. injection of 50 mg/kg sodium pentobarbital and blood was collected by cardiac puncture using an EDTA-coated syringe. Tumors and adipose tissues from mammary and abdominal fat depots were collected and frozen at −80 °C until analysis. Samples of the tumors and adipose tissues were also fixed in 10% formalin for immunohistochemistry. A total of five to six mice were used per experimental group. Further details of the mice used in different experiments are also described in the figure legends.

### 4.4. Measurements of the Effects of DEX on the RT-Induced Fibrosis in Normal Mice

For determining the effects of DEX on RT-induced fibrosis, normal Balb/c mice were irradiated in the left 2nd mammary fat pad using the SARRP with 5 daily fractions of 7.5 Gy. Mice were treated with 3 mg DEX/kg i.p. or vehicle [41] at 1 day before RT, during 5 daily fractions of RT and for 3 days after RT. Mice were anesthetized terminally at 7 weeks after completion of the RT and plasma, adipose tissue and lungs were collected and stored at −80 °C. Samples of adipose tissue and lungs underlying the fat pad were also fixed in 10% formalin for histochemistry. A total of six mice were used per experimental group. Further details of the mice used in different experiments are described in the figure legends.

### 4.5. Multiplex Analysis of Cytokines and Hormones

Centrifuged culture media from human adipose tissue were analyzed for cytokines and chemokines using the Human Cytokine/Chemokine 65-plex ELISA Discovery Luminex assay by Eve Technologies Corp (Calgary, AB, Canada). Mouse plasma and tissue homogenates were analyzed for cytokines/chemokines and hormones with Mouse Cytokine/Chemokine 32-plex ELISA and Mouse Metabolic 11-plex arrays by Eve Technologies Corp. Concentrations obtained from the assay were normalized to both the volume of medium and protein concentration of the samples as determined by the BCA protein assay (Thermo Fisher Scientific, Rockford, IL, USA).

### 4.6. Measurement of ATX Activity

ATX activity was measured in culture medium or in mouse plasma and tissues as described previously based on choline release from lysophosphatidylcholine [41]. For detecting ATX activity in the adipose tissue, tissue homogenates were mixed with the same volume of 8 mM C14:0-lysophosphatidylcholine in buffer A (100 mM Tris-HCl, pH 9.0; 500 mM NaCl; 5 mM MgCl_2_; and 0.05% v/v Triton X-100) and incubated for 3 h at 37 °C. For detecting ATX activity in plasma containing EDTA as an anticoagulant, the assay buffer A was supplemented with 25 mM CaCl_2_ and 25 mM MgCl_2_. After the incubation, 20-μL samples were mixed with 90 μL of buffer C containing 88 μL of buffer B (100 mM Tris-HCl, pH 8.5, and 5 mM CaCl_2_), 0.7 μL of 10 mM Amplex^TM^ Red, 0.1 μL of 1000 U/mL horseradish peroxidase, and 1.2 μL of 300 U/mL choline oxidase. Choline formation was measured at λ_ex_ = 544 nm/λ_em_ = 590 nm using a Fluoroskan Ascent™ FL Fluorometer (Thermo Fisher Scientific, Vantaa, Finland).

### 4.7. Quantitative Real-Time PCR (qRT-PCR)

RNA was isolated using Trizol^®^ (Invitrogen Life Technologies, Carlsbad, CA, USA) with the Direct-zol™ RNA MiniPrep kit (Zymo Research, Irvine, CA, USA) according the manufacturer’s instructions. qRT-PCR was then performed using primers as described [41]. Quantitative RT-PCR analysis was performed using RT^2^ SYBRgreen quantitative PCR mastermix (Qiagen, Montréal, PQ, Canada) in the Applied Biosystems 7500 real-time RT-PCR system (Life Technologies, Grand Island, NY, USA). The relative abundance of target mRNA was determined from cycle threshold values (Ct) and normalized to the geometric mean of the housekeeping gene cyclophilin A. 

### 4.8. Immunohistochemistry and Western Blots

Tumors, adipose tissues and lung were fixed with 10% formalin followed with paraffin embedding and sectioning. Sample treatment and immuno-staining were performed according to a standard procedure [84]. Antigen retrieval was performed by heating with Tris/EDTA (pH 9.0) in a pressure cooker. For collagen detection, paraffin-embedded sections of adipose tissue and lungs were stained with 0.1% Sirius Red solution (0.5 g Sirius Red dissolved in saturated picric acid aqueous solution) for 1 h and washed twice with 0.5% acetic acid. Trichrome staining was performed using the Trichrome Stain Kit (ab150686) from Abcam (Cambridge, UK) according to the manufacturer’s instructions. Images were acquired using a Zeiss Axioskop 2 imaging system (Carl Zeiss Canada, Toronto, ON, Canada). The average counts in 5 fields were calculated for each sample. Specific proteins were measured by Western blot as described previously [21]. Immunoblots were analyzed by the LI-COR Odyssey Imaging System (LI-COR Biosciences, Lincoln, NB, USA).

### 4.9. Statistics

Results were expressed as means ± SEM. Statistical significance between paired groups was determined using a paired t-test. A one- or two-way ANOVA with a Bonferroni post hoc test was used for multiple comparisons according to the experiment design using SPSS 16.0 software (SPSS Inc., Chicago, IL, USA). *p* < 0.05 was considered statistically significant. 

## 5. Conclusions

Multiple fractions of RT to the whole breast after lumpectomy should stimulate ATX secretion from adipocytes, promoting LPA production and signaling through the concomitantly increased levels of LPA_1_ receptors after RT [39]. These effects of LPA activate the inflammatory cycle and likely drive the progression of fibrosis.

## Supplementary Materials

The following are available online at https://www.mdpi.com/2072-6694/12/4/999/s1, Figure S1: More complete Western blots for Figure 2, Figure 3 and Figure 8 and Figure S2: DEX did not change ATX activity in either normal mice or tumor-bearing mice at 7 weeks after RT.

## Abbreviations

ATX	autotaxin
BLM	bleomycin
COX-2	cyclooxygenase-2
DEX	dexamethasone
CCL	C-C motif chemokine ligand
CXCL	C-X-C motif chemokine ligand
G-CSF	granulocyte-colony stimulating factor
GCLC	glutamate-cysteine ligase catalytic subunit
IL	interleukin
LPA	lysophosphatidic acid, which at physiological pH is in a salt form, lysophosphatidate
LPAR_1,2_	LPA receptors types 1− and 2
LPP	lipid phosphate phosphatase
Nrf2	nuclear erythroid 2-like 2
RT	radiation therapy
TGF-β	transforming growth factor β
TNFα	tumor necrosis factor-α
VEGF	vascular endothelial growth factor
