# Supplementary material for: Dexamethasone Attenuates X-Ray-Induced Activation of the Autotaxin-Lysophosphatidate-Inflammatory Cycle in Breast Tissue and Subsequent Breast Fibrosis
Source: Cancers (Basel). 2020 Apr 18;12(4):999. doi: 10.3390/cancers12040999 (PMC7226295; doi:10.3390/cancers12040999)
Supplement: Supplementary file 1 [file cancers-12-00999-s001.pdf]

Supplementary Materials: Dexamethasone Attenuates X-ray-Induced Activation of the Autotaxin-Lysophosphatidate-Inflammatory Cycle in Breast Tissue and Subsequent Breast Fibrosis

Guanmin Meng, Melinda Wuest, Xiaoyun Tang, Jennifer Dufour, Todd P.W. McMullen, Frank Wuest, David Murray and David N. Brindley

A)

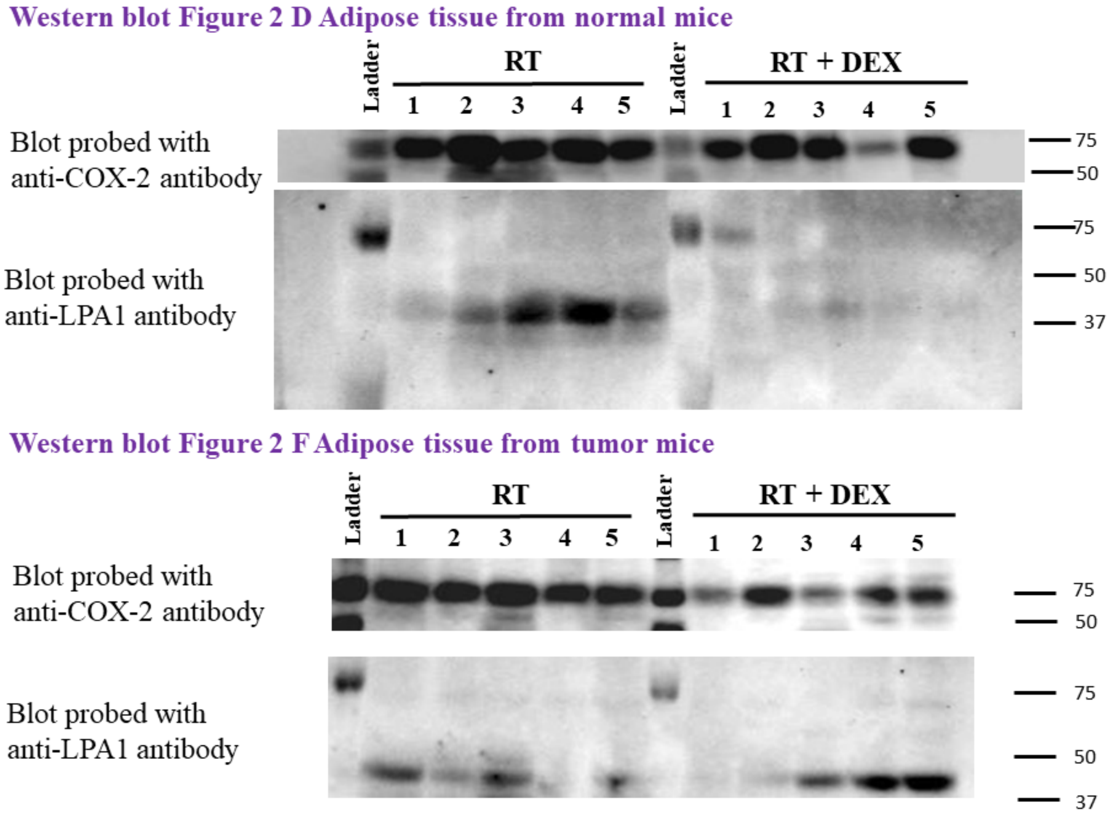

B)

Western blot Figure 3C

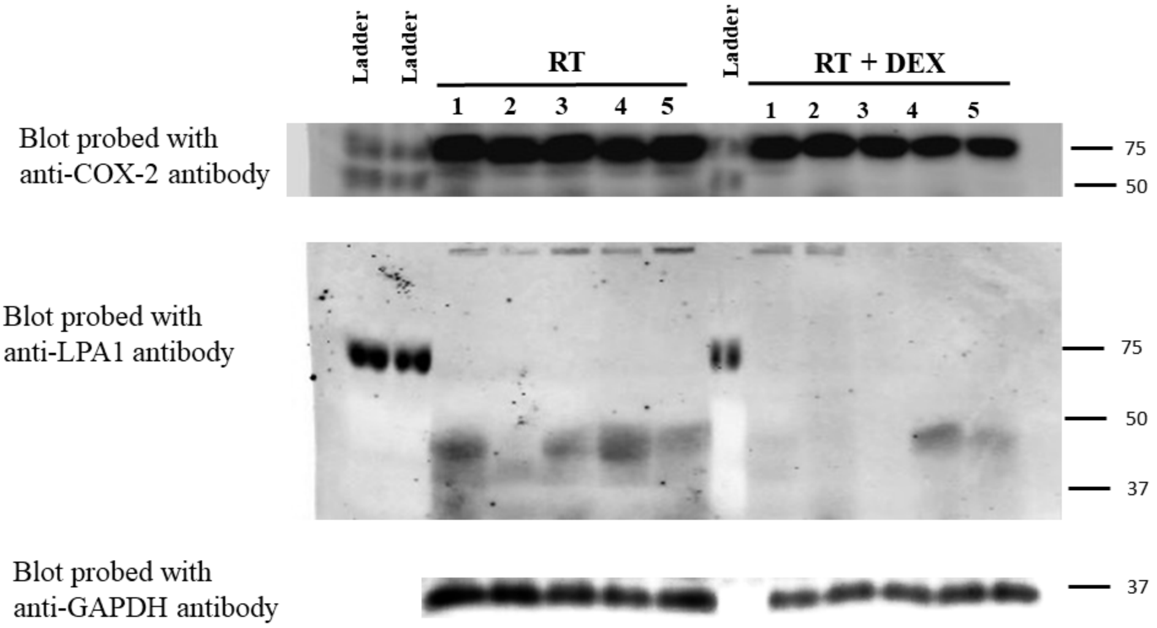

C)

Western blot Figure 8

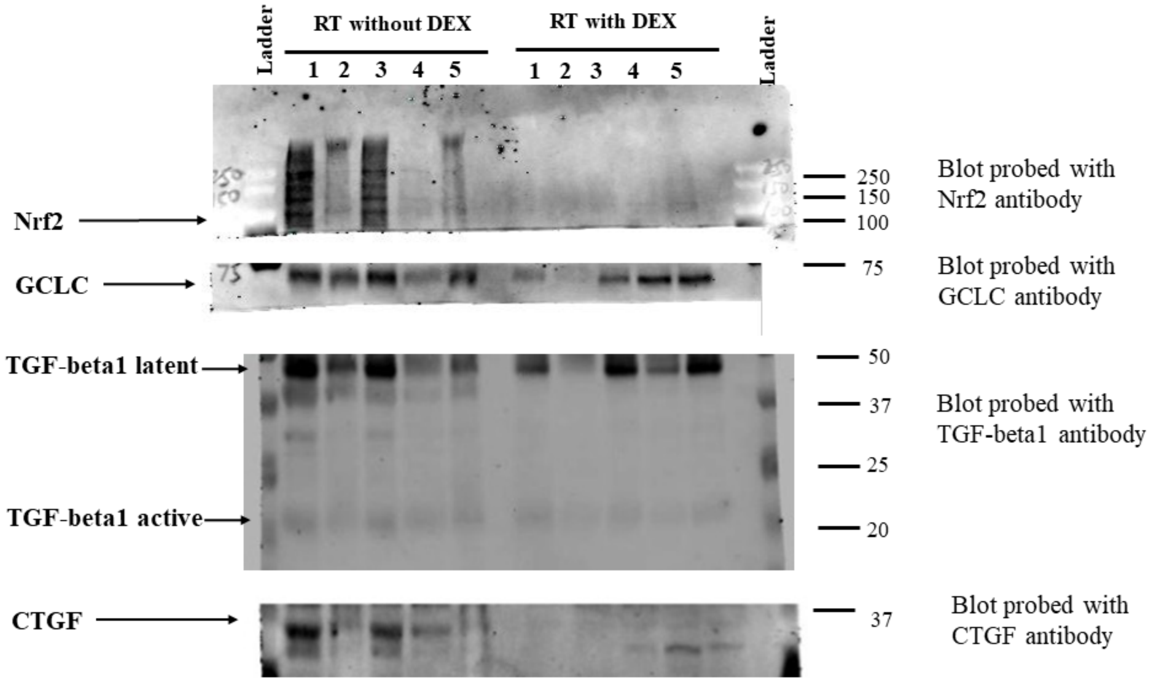

Figure S1. More complete Western blots for Figures 2, 3 and 8. The original membranes from Figure 2 (A), Figure 3 (B) and Figure 8 (C) as shown were cut according to the molecular weight markers and were then probed with the appropriate antibodies as shown in examples.

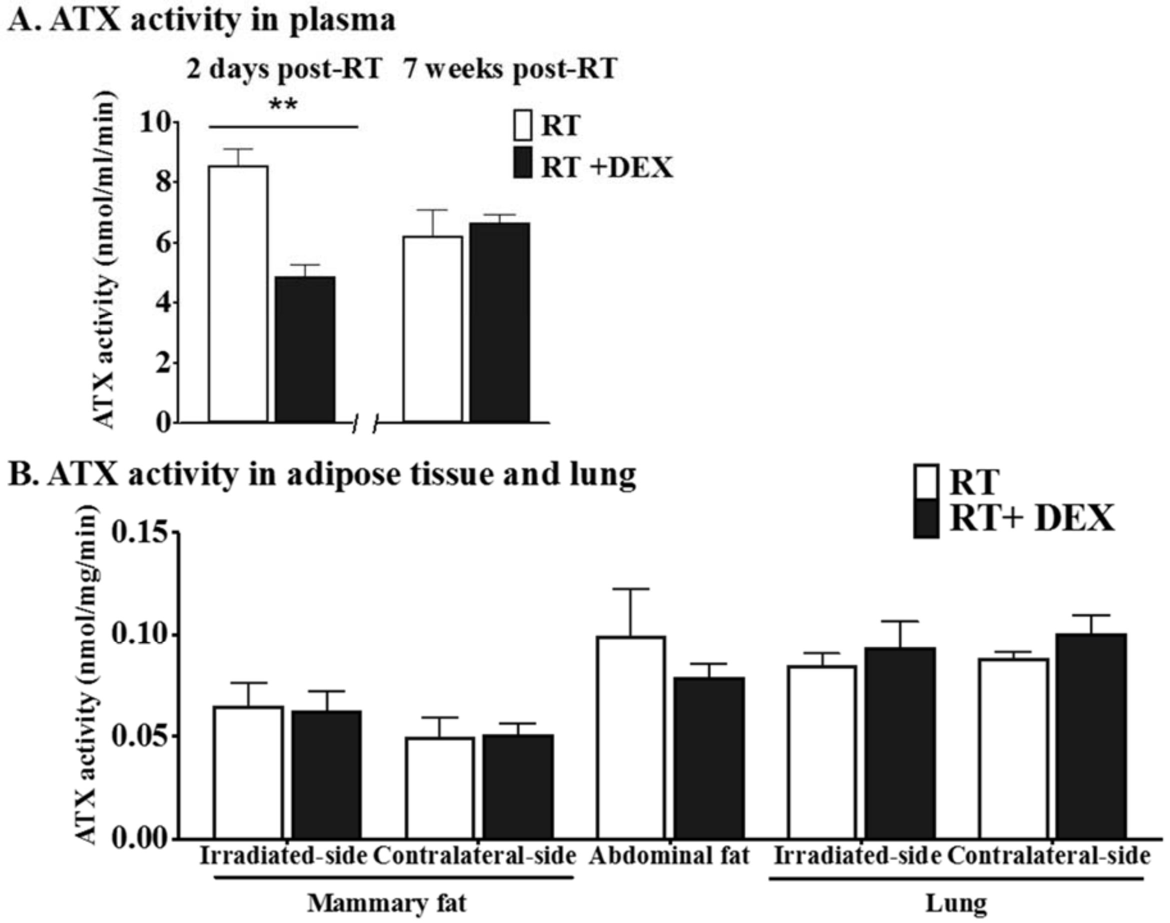

Figure S2. DEX did not change ATX activity in either normal mice or tumor-bearing mice at 7 weeks after RT. Mice were treated daily with DEX (3 mg/kg/day) or vehicle at 1 day before RT, during 5 daily 7.5-Gy fractions of RT to an upper breast fat pad, and for 3 days after RT. At 7 weeks after completion of the RT, the ATX activity in plasma (**A**) and adipose tissues from mammary and abdominal fat depots and lung (**B**) were determined. Results are expressed as means ± SEM from 6 mice per group. ** $p < 0.01$.

© 2020 by the authors. Licensee MDPI, Basel, Switzerland. This article is an open access article distributed under the terms and conditions of the Creative Commons Attribution (CC BY) license (http://creativecommons.org/licenses/by/4.0/).
